# LncRNA PCAT6 promotes proliferation, migration, invasion, and epithelial-mesenchymal transition of lung adenocarcinoma cell by targeting miR-545-3p

**DOI:** 10.1007/s11033-023-08259-x

**Published:** 2023-02-14

**Authors:** Chuyi Yang, Hongyu Huang, Yongpeng Li, Ting Zhuo, Lu Zhu, Chenyang Luo, Yanbin Wu, Shouming Qin

**Affiliations:** 1 Department of Pulmonary and Critical Care Medicine, The First Affiliated Hospital of Guangxi Medical University, Nanning, China; 2 Department of the urology, Guangxi Medical University Cancer Hospital, Nanning, China

**Keywords:** lncRNA PCAT6, miR-545- 3p, Lung adenocarcinoma, EMT

## Abstract

**Background:**

Lung cancer is a high incidence cancer on a worldwide basis and has become a major public health problem. Lung adenocarcinoma (LUAD) makes up approximately half of all lung cancers and is a threat to human health. Long non-coding RNAs (lncRNAs) is an important regulator of the development and progression of lung adenocarcinoma. In this manuscript we examined the role and potential mechanism of lncRNA PCAT6 in the development of LUAD.

**Methods and results:**

Differences in lncRNA PCAT6 levels between LUAD samples and normal samples were first explored in the GEPIA database. We found that lncRNA PCAT6 expression was elevated, which was also validated in lung adenocarcinoma tissues and cell lines. Using western blotting, CCK-8, EdU, wound healing and transwell assays, we found that knockdown of lncRNA PCAT6 inhibited EMT, proliferation, migration, and invasion of LUAD cells. We noted a predicted a binding site for lncRNA PCAT6 and miR-545-3p through conducting bioinformatic analyses, and their binding was subsequently verified by a dual-luciferase reporter assay. Rescue experiments confirmed that miR-545-3p inhibitor partially abolished the inhibition function of lncRNA PCAT6 knockdown on LUAD cells. In addition, we predicted the downstream target genes of miR-545-3p and verified them by RT-qPCR. We found that EGFR was reduced in the silence of lncRNA PCAT6 and upregulated after miR-545-3p inhibition.

**Conclusion:**

This study demonstrates that lncRNA PCAT6 promotes a more aggressive LUAD phenotype by sponging miR-545-3p. This finding may provide new ideas for the treatment of lung cancer.

**Supplementary information:**

The online version contains supplementary material available at 10.1007/s11033-023-08259-x.

## Introduction

Lung cancer currently remains the most prevalent malignant diseases worldwide and has serious public health implications [1, 2]. Early symptoms of lung cancer are relatively benign, and this contributes to low detection rates in early disease stages. The metastatic rate of advanced lung cancer is high and the overall prognosis is poor. The overall 5-year mortality rate for lung cancer is still high at approximately 78% [1, 3]. Lung adenocarcinoma (LUAD) is a malignant epithelial tumor with adenoid differentiation and/or mucous secretion and comprises about 40% of all lung cancers [4, 5]. At, present, the pathogenesis of LUAD has not been fully elucidated; consequently, it is vital to further understand mechanisms of LUAD development to uncover novel targets to improve the diagnosis, prevention, and therapy of LUAD.

Long non-coding RNAs (lncRNAs) are a class of non-coding RNA transcripts that possess a length of more than 200 nucleotides [6]. LncRNAs are involved in regulating gene expression in multiple dimensions such as epigenetic regulation, chromatin modifications, transcription, and post-transcription regulation. LncRNAs affect tumor growth, apoptosis, invasion, metastasis, and the epithelial-mesenchymal transition (EMT) [7, 8]. The role of some lncRNAs such as HOTAIR, MALAT1, H19, ANRIL [9] in LUAD has been studied, but there are unknown lncRNAs that contribute to cancer pathogenesis and their mechanism of action requires further investigation.

LncRNA prostate cancer-associated transcript 6 (PCAT6) was first discovered as a prostate cancer regulatory component and located on 1q32.1 [10]. Subsequently, many studies have demonstrated that lncRNA PCAT6 expression is elevated in various tumors and that this may be linked to tumor progression [11]. One study found that lncRNA PCAT6 promoted growth, invasion, and migration of A549 and H1975 cells via regulation of miR-330-5p [12]. Additional studies proved that lncRNA PCAT6 enhanced cell growth and EMT by targeting miR-15a [13]. The up-regulated expression of lncRNA PCAT6 can promote cell growth, migration, and invasion, indicating that lncRNA PCAT6 has oncogenic functions. However, how lncRNA PCAT6 contributes to the progress of LUAD remains to be studied.

MicroRNAs (miRNAs) are conserved, non-coding short RNA molecules [14, 15]. MiRNAs can regulate numerous cellular functions such as cell growth, migration, invasion, and EMT [16]. Significant evidence shows that abnormal regulation of miRNAs is related to the malignant progression of many cancers, and miRNAs are potential therapeutic targets for lung cancer [17]. For example, miR-545-3p has been studied in a variety of cancers including endometrial [18] and ovarian cancer [19]. Another study showed that miR-545-3p was expressed at low levels within NSCLC tumor and cultured cells [20]. In view of the important function of miR-545-3p in lung cancer, and bioinformatics analyses outlined in this work, we hypothesize that lncRNA PCAT6 may exert its physiological function by targeting miR-545-3p. However, regulatory networks linking miR-545-3p and lncRNA PCAT6 remain to be studied.

In the present study, we studied the effect and mechanism of lncRNA PCAT6 in LUAD. We document that lncRNA PCAT6 is up-regulated and pro-carcinogenic in LUAD. Further, silencing lncRNA PCAT6 has an anti-tumorigenic effect. Our investigation suggested that lncRNA PCAT6 plays a role in LUAD pathogenesis by targeting miR-545-3p and we subsequently investigated the molecular mechanism controlling this effect.

## Materials and methods

### Patient samples

We obtained 25 LUAD samples and their surrounding normal samples (about 5 cm from the tumor) from the First Affiliated Hospital of Guangxi Medical University that were collected from 2019 to 2020. None of these patients had been treated for their cancer prior to surgery. The collected tissue was placed in liquid nitrogen following procurement. All patients provided informed consent and this study was approved by the Ethics Committee of the First Affiliated Hospital of Guangxi Medical University.

### Cell culture

Wuhan Procell Life Science &Technology Co., Ltd provided the A549 and H1975 LUAD cell lines and human normal lung epithelial cells BEAS-2B. LUAD H1299 cells were provided by Chinese Academy of Sciences (Shanghai). A549 and BEAS-2B cells were cultured in DMEM (Gibco) supplemented with 10% fetal bovine serum (FBS, Gibco). H1299 and H1975 cells were cultured in RPMI-1640 medium (Gibco) supplemented with 10% FBS. The medium was changed once daily with fresh medium and dissociated with 0.25% trypsin (Gibco) when cell density reached 90%. All cells were placed in a humidified incubator at 37 °C containing a 5% CO2 atmosphere.

### Cell transfection

Three small interfering RNA (siRNA) sequences derived from the lncRNA PCAT6 transcript were synthesized by Guangzhou RiboBio Biology Co., Ltd. The sequences of the siRNAs are: si-PCAT6 #1 5’-UGGCCUAGGAACCCGAACCUGACCC-3’; si-PCAT6 #2 5’-AAACAUUCCAGGGCACCGAGAGAUG-3’; and si-PCAT6 #3 5’-GGTGTCTCCATCCTCATTC-3’. MiR-545-3p mimic, miR-545-3p inhibitor, and their corresponding controls were purchased from Guangzhou RiboBio Co., Ltd. Subsequently, we transfected these oligonucleotides into LUAD cells using lipofectamine 3000 (L300015, Thermo).

### RNA isolation and RT-qPCR

Total RNA was isolated using TRIzol reagent (Takara). RNA samples were quantified using a Nanodrop ND2000 (Thermo Science). RNA samples with an OD 260/280 value in the range of 1.8-2.0 were transcribed into cDNA using a reverse transcription kit (Takara). Real-time quantitative PCR (RT-qPCR) was conducted using a SYBR Green PCR kit (Takara) and a LightCycler480 System (Roche Ltd.) instrument. GAPDH or U6 transcripts were used as internal controls to quantify relative transcript abundance. All data were analyzed using the 2 − ΔΔCt method. RT-qPCR for lncRNA PCAT6 was conducted with the forward primer 5′-CCCCTCCTTACTCTTGGACAAC-3′ and reverse primer 5′-GACCGAATGAGGATGGAGACAC-3′. GAPDH primers used were forward: 5′- AGAAGGCTGGGGCTCATTTG-3′ and reverse: 5′-AGGGGCCATCCACAGTCTTC-3′. U6 primers used were forward: 5′-GCTTCGGCAGCACATATACTAAAAT-3′ and reverse: 5′-CGCTTCACGAATTTGCGTGTCAT − 3′.

### Cytoplasmic-nuclear fractionation

Cytoplasmic and nuclear RNA were isolated from two LUAD cell lines using the Cytoplasmic & Nuclear RNA Purification kit (Cat.21,000; Norgen). First, we split the cells on ice with a lysis buffer for 5 min and after the lysate was centrifuged at 14,000 g for 10 min and was subsequently decanted into a new tube. Cold cell separation buffer was then added to separate the nucleus and cytoplasmic fractions, and subsequently cytoplasmic and nuclear RNA were obtained. RT-qPCR was used to quantify indicated transcripts. GAPDH was used as the internal control for the cytoplasmic RNA fraction while the U6 was used for the nuclear RNA fraction.

### Dual-luciferase reporter assay

In order to examine lncRNA PCAT6 association with miR-545-3p, lncRNA PCAT6 wild type and lncRNA PCAT6 mutant sequences were subcloned into the pmiR-Rb-Report^™^ Vector (Guangzhou RiboBio Co., Ltd.). Per the manual, constructs encoding miR-545-3p-mimic, miR-normal control, as well as lncRNA PCAT6 wildtype or PCAT6 mutant sequences were co-transfected into HEK 293T cells using Lipofectamine 3000. Luciferase activity was measured using a dual-luciferase reporter system (Promega) and firefly luciferase activity was normalized to Renilla luciferase activity.

### Cell counting kit-8 (CCK-8)

LUAD cells transfected with si-PCAT6 for 24 h were seeded into 96-well plates at an adjusted density of 1 × 10^3^ per well, and placed in a CO2 incubator. Cell viability was determined via CCK-8 kit (Beyotime). At 0, 24, 48 and 72 h, 10 µL of CCK-8 reagent was added to each well. After that, we cultured them in incubator for 2 h and detected the absorbance at 450 nm.

### EdU assay

We measured cell proliferation using the BeyoClickTMEdU-555 Cell Proliferation Detection kit (Beyotime). Indicated LUAD cells were grown in 6-well plates and transfected using Lipofectamine 3000 when the culture density reached approximately 50%. Twenty-four hr later, 20 mM EdU reaction solution (C0075S; Beyotime) was added to each well and the plate subsequently placed back into the incubator for 2 h. LUAD cells were subsequently fixed with Immunol Staining Fix Solution (P0098; Beyotime) and then stained with Hoechst 33,342 (1:1000). Finally, cells were visualized and photographed using a fluorescence microscope.

### Wound healing assay

LUAD cells were seeded into a six-well plate with horizontal lines drown on the bottom surface of the plate. After the cell density reaches about 80–90%, wounds are made on the monolayer with a sterile tip of 10 µl perpendicular to the line. We used PBS to wash the cells and incubated them in serum-free medium for 24 h. Finally, images of the same area were taken with a microscope at 0 and 24 h after scratching the cell layer.

### Transwell assay

Transwell migration and invasion experiments were carried out using a transwell chamber with 8 μm pore size (Corning), that were either uncoated or pre-coated with Matrigel Matrix (BD Bioscience, USA). A549 and H1299 cells were transfected for 24 h, digested with trypsin, harvested by centrifugation, and cells suspended in FBS-free medium at a final density of 5 × 10^4^ cells/ml. Subsequently, 250 µL of cell suspension was added to the upper chamber and 700 µL of medium containing 10% FBS was added to the lower chamber. The plates were subsequently placed in an incubator for 36 h after which cells in the lower chamber were fixed with 4% formaldehyde for 20 min, and stained with 0.5% crystal violet (Beyotime) for 15 min. Cell numbers were counted in several randomly chosen areas using a light microscope (Olympus).

### Western blotting assay

A549 and H1299 cells were harvested 72 h after transfection and lysed with cold RIPA lysis buffer (Beyotime) containing PMSF for 20 min on ice. After centrifugation at 14,000 x g for 10 min, the supernatant was decanted into a new tube. Subsequently, we used a BCA protein assay kit (Solarbio) to measure the protein concentration. 40 µg of protein and 5ul of protein marker were resolved by 7% SDS-PAGE and the gel contents electrotransferred onto PVDF membranes. After incubation in 5% non-fat milk (Solarbio) for 2 h, the membrane and primary antibodies were incubated, with rocking, overnight at 4 °C. After TBST washes, PVDF membranes and corresponding secondary antibodies (ABMARK) were incubated at 37 °C for 2 h. Finally, we used an ECL kit (Beyotime) to visualize protein bands on the membrane. Assessment of protein abundance was determined using Image J software.

### Statistical analysis

Data were quantified and presented as mean ± standard deviation (SD) using GraphPad Prism 8.0 and SPSS23.0 software. Comparisons among two independent samples were performed using t-tests. In addition, paired t-tests were used to compare between LUAD tissue and precancerous tissue. When comparing data between multiple groups, we used one-way ANOVA. Differences were considered statistically significant at p < 0.05. Every experiment was repeated more than three times.

## Results

### LncRNA PCAT6 is upregulated in cultured LUAD cells and tissues

**Fig. 1:**
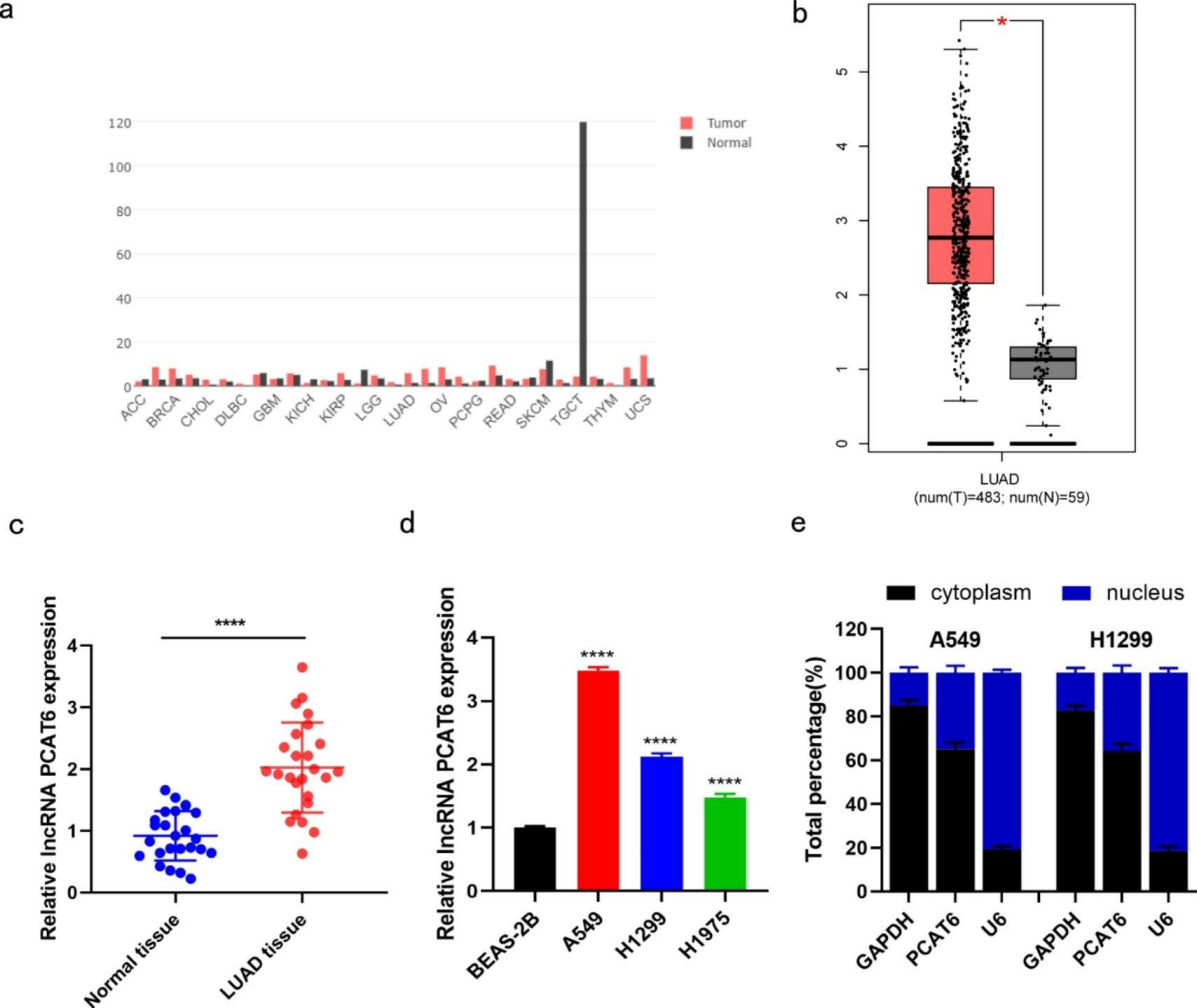
LncRNA PCAT6 was highly expressed in lung adenocarcinoma (a-b) GEPIA database showed abnormal expression of lncRNA PCAT6 in various tumors, and increased expression level in LUAD. (c) The expression level of lncRNA PCAT6 in lung adenocarcinoma samples and normal adjacent samples was detected by RT-qPCR. (d) The expression levels of lncRNA PCAT6 in three LUAD cell lines (A549, H1299 and H1975) and healthy BEAS-2B cells were detected by RT-qPCR. (e) Cytoplasmic-Nuclear fractionation was used to detect the proportion of PCAT6 in nucleus and cytoplasm. * p < 0.05, ** p < 0.01, *** p < 0.001

Using the GEPIA database (http://gepia.cancer-pku.cn/detail.php), we found that lncRNA PCAT6 was abnormally expressed in some malignant tumors and highly expressed in LUAD tissues (Fig. 1a-b). To verify this finding, we used RT-qPCR to detect lncRNA PCAT6 expression in 25 LUAD tumor and adjacent normal tissue samples. Compared with normal adjacent tissue, lncRNA PCAT6 was highly expressed in LUAD samples (Fig. 1c). In comparison to non-tumorigenic BEAS-2B cells, lncRNA PCAT6 expression was prominently increased in A549, H1299 and H1975 cells. Since lncRNA PCAT6 was more highly expressed in H1299 and A549 cells, we chose these two cell lines for subsequent experiments (Fig. 1d). We examined the location of lncRNA PCAT6 within these cells. Cytoplasmic-Nuclear fractionation indicated that lncRNA PCAT6 was expressed in both cytoplasmic and nuclear RNA pools, and that cytoplasmic abundance was higher than the nuclear fraction (Fig. 1e). Taken together, these results indicate that lncRNA PCAT6 is highly expressed in LUAD tissues and cultured cell lines. Thus, lncRNA PCAT6 has potential to be a valid tumor marker for LUAD.

### Knockdown of lncRNA PCAT6 reduces growth, invasion, migration, and EMT in cultured LUAD cells

**Fig. 2:**
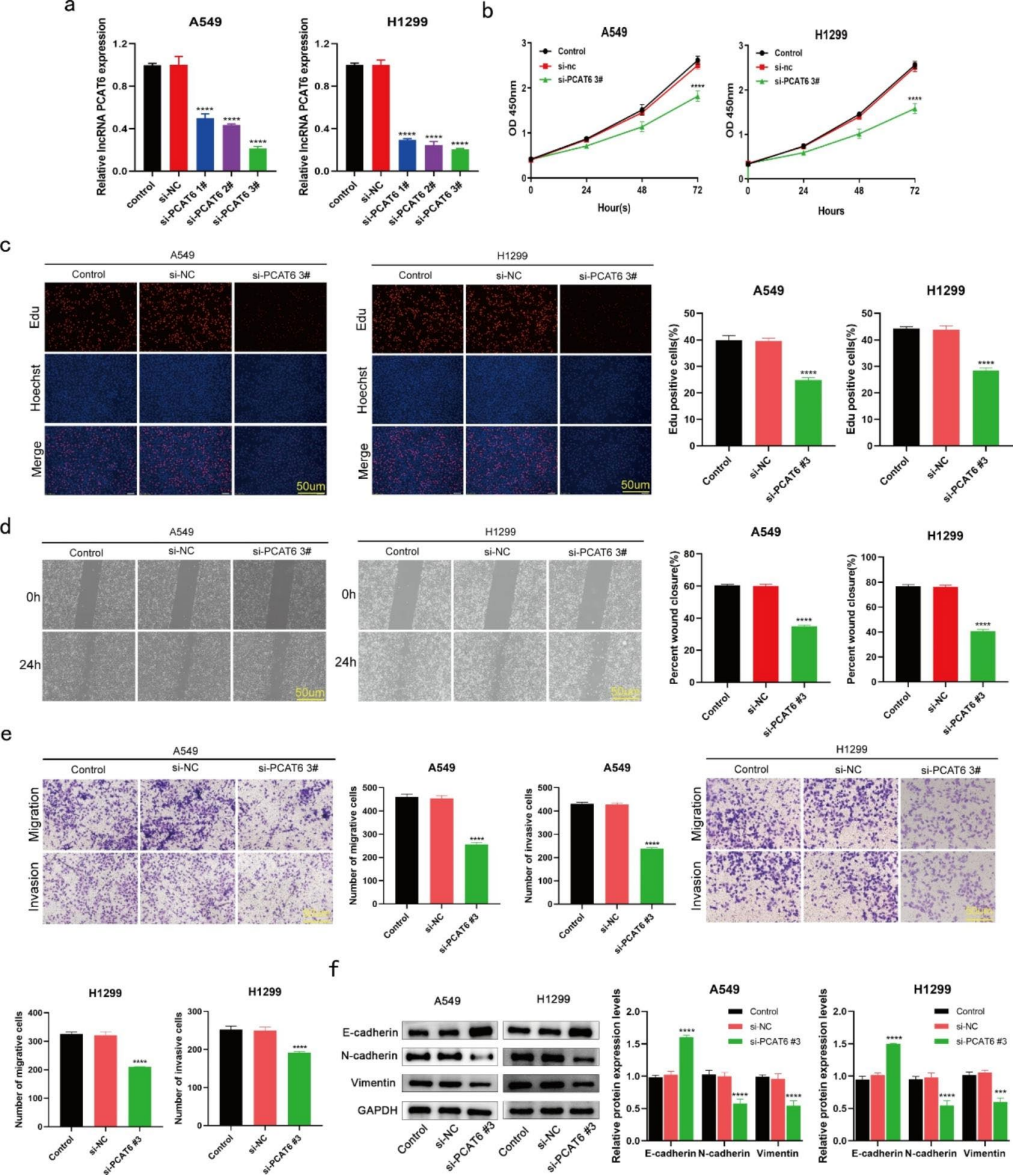
Silencing of lncRNA PCAT6 inhibited LUAD cell growth, migration, invasion and EMT (a) RT‑qPCR analysis of the transfection efficiency of three si-PCAT6 sequences. (b-c) EdU assays and CCK8 were performed to examin A549 and H1299 cell growth. (d-e) Scratch assays and transwell assays were performed to examine the effect of silencing of PCAT6 on LUAD cells. (f) WB assays tested the protein levels of EMT-related protein. *P < 0.05, **P < 0.01, ***P < 0.001 vs. si-NC group

To further explore the role of lncRNA PCAT6 in LUAD, we downregulated lncRNA PCAT6 by transfecting cells with three independent si-PCAT6 sequences. We first demonstrated transfection efficiency using RT-qPCR (Fig. 2a). The data revealed that si-PCAT6 #3 exerted the most significant knockdown effect, so we chose si-PCAT6 #3 sequence for subsequent experiments. CCK-8 and EdU experiments indicated that lncRNA PCAT6 negatively influenced LUAD cell growth as we observed that silencing of lncRNA PCAT6 markedly reduced rates of cell growth (Fig. 2b-c). Subsequently, cell scratch (Fig. 2d) and transwell assays (Fig. 2e) revealed that knockdown of lncRNA PCAT6 greatly reduced migration and invasion, respectively, in comparison to cells transfected with a normal control (NC) siRNA. Additionally, after lncRNA PCAT6 knockdown, we observed that E-cadherin protein levels were greatly elevated, whereas N-cadherin and Vimentin protein levels were significantly decreased (Fig. 2f). Overall, the findings demonstrated that knockdown of lncRNA PCAT6 restrains cultured LUAD cell EMT, growth, migration and invasion characteristics.

### MiR-545-3p is a target of lncRNA PCAT6 and is negatively correlated with lncRNA PCAT6 expression

**Fig. 3:**
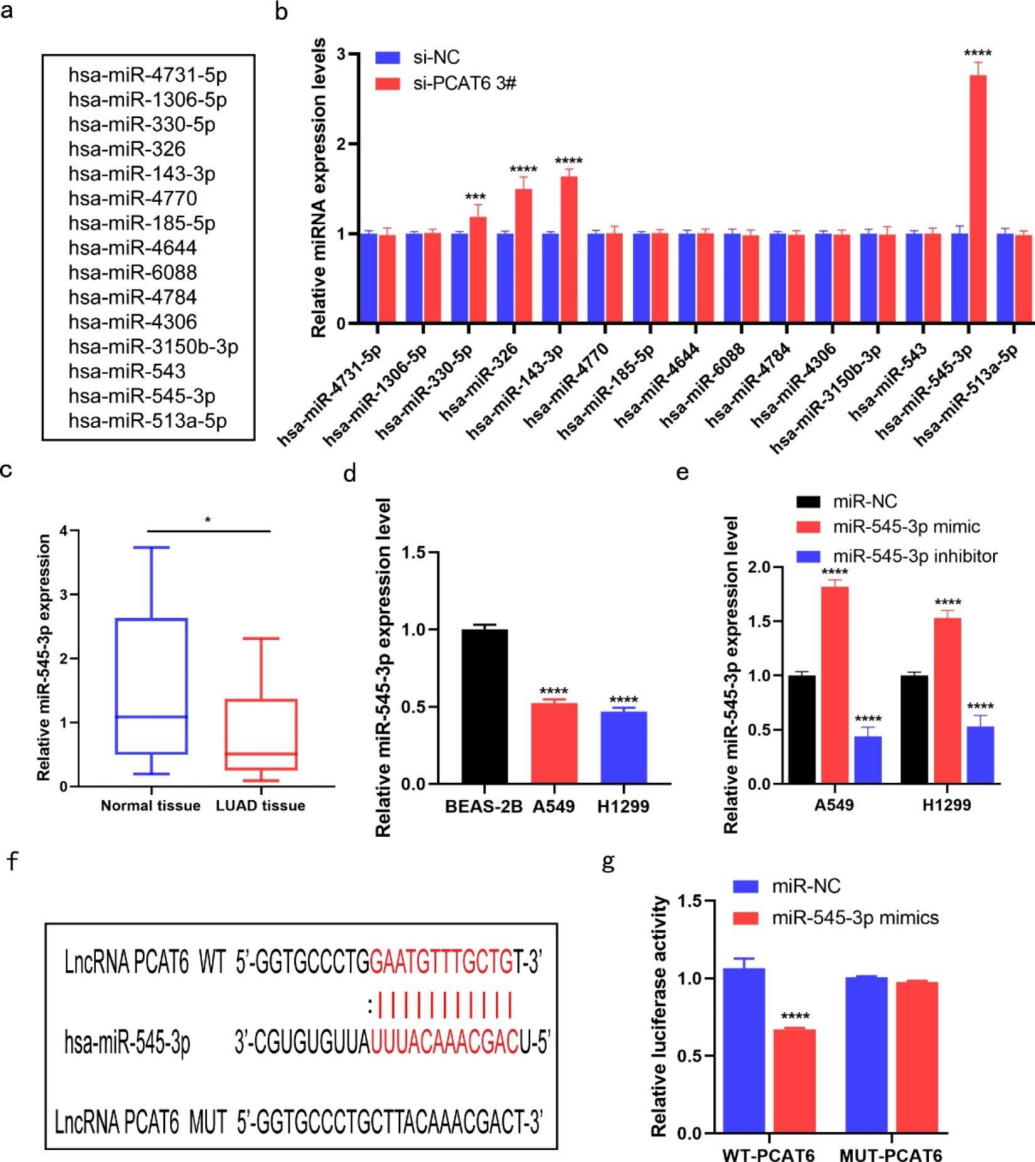
MiR-545-3p can target lncRNA PCAT6 gene and negatively regulate its expression (a) The targeted miRNAs of PCAT6 were predicted by StarBase database. (b) RT-qPCR was used to detect the association between miRNAs and PCAT6. (c-d) The expression of miR-545-3p in LUAD samples and cells was observed by RT-qPCR. (e) Detection of miR-545-3p mimics and inhibitors by RT-qPCR. (f) The binding sites between PCAT6 with miR-545-3p. (g) Predicted spots were detected by dual-Luciferase reporter assay. *P < 0.05, **P < 0.01, ***P < 0.001

The StarBase database (http://starbase.sysu.edu.cn/) was utilized to predict potential target genes for lncRNA PCAT6 (Fig. 3a). Results from this investigation showed that a total of 15 miRNAs were predicted to be the downstream miRNA targets of lncRNA PCAT6. Subsequently, we transfected cells and analyzed changes in these miRNAs. RT-qPCR experiments indicated that expression of miR-545-3p was remarkably increased in these miRNAs (Fig. 3b). In addition, miR-545-3p showed reduced expression in LUAD tissue compare to normal adjacent tissue samples (Fig. 3c). In comparison to BEAS-2B cells, miR-545-3p abundance was greatly reduced in both H1299 and A549 cells (Fig. 3d). We next assessed the transfection efficiency of miR-545-3p mimics and inhibitors by RT-qPCR (Fig. 3e). To explore whether lncRNA PCAT6 targets miR-545-3p, we used bioinformatics to predict a potential binding site for miR-545-3p and lncRNA PCAT6 interaction (Fig. 3f). The results of the dual luciferase reporter assay showed a decrease in luciferase activity of lncRNA PCAT6-wildtype but not lncRNA PCAT6-mutant constructs after the introduction of miR-545-3p mimics into the cells (Fig. 3 g). This result indicates that miR-545-3p abundance has a reciprocal relationship with lncRNA PCAT6 and suggests that this is controlled by miR-543-3p binding to the 3ʹUTR of PCAT6. In sum, we conclude that miR-545-3p is a target of lncRNA PCAT6.

### Inhibition of miR-545-3p reversed the effects of lncRNA PCAT6 knockdown on cell proliferation, migration, invasion and EMT

**Fig. 4:**
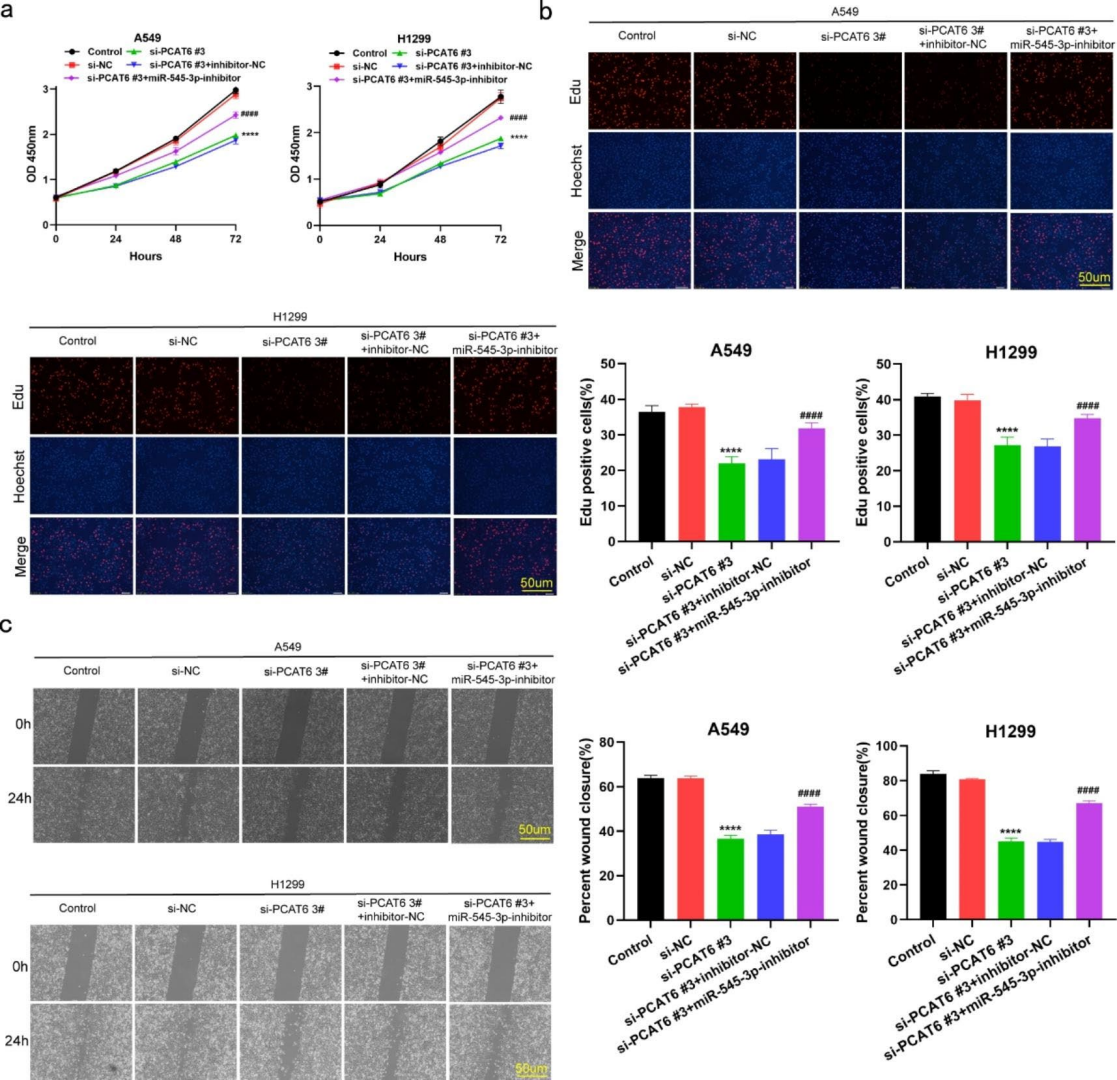
MiR-545-3p inhibitor partially reversed lncRNA PCAT6 silencing’s suppression on LUAD cell growth and migration (a-b) H1299 and A549 cell proliferation measured through EdU and CCK8 assays; (c) Scratch assays conducted to examine H1299 and A549 cell migration. *P < 0.05, **P < 0.01, ***P < 0.001 vs. si-NC group; #P < 0.05, ##P < 0.01, ###P < 0.001 vs. si-PCAT6#3 + inhibitor-NC group

**Fig. 5:**
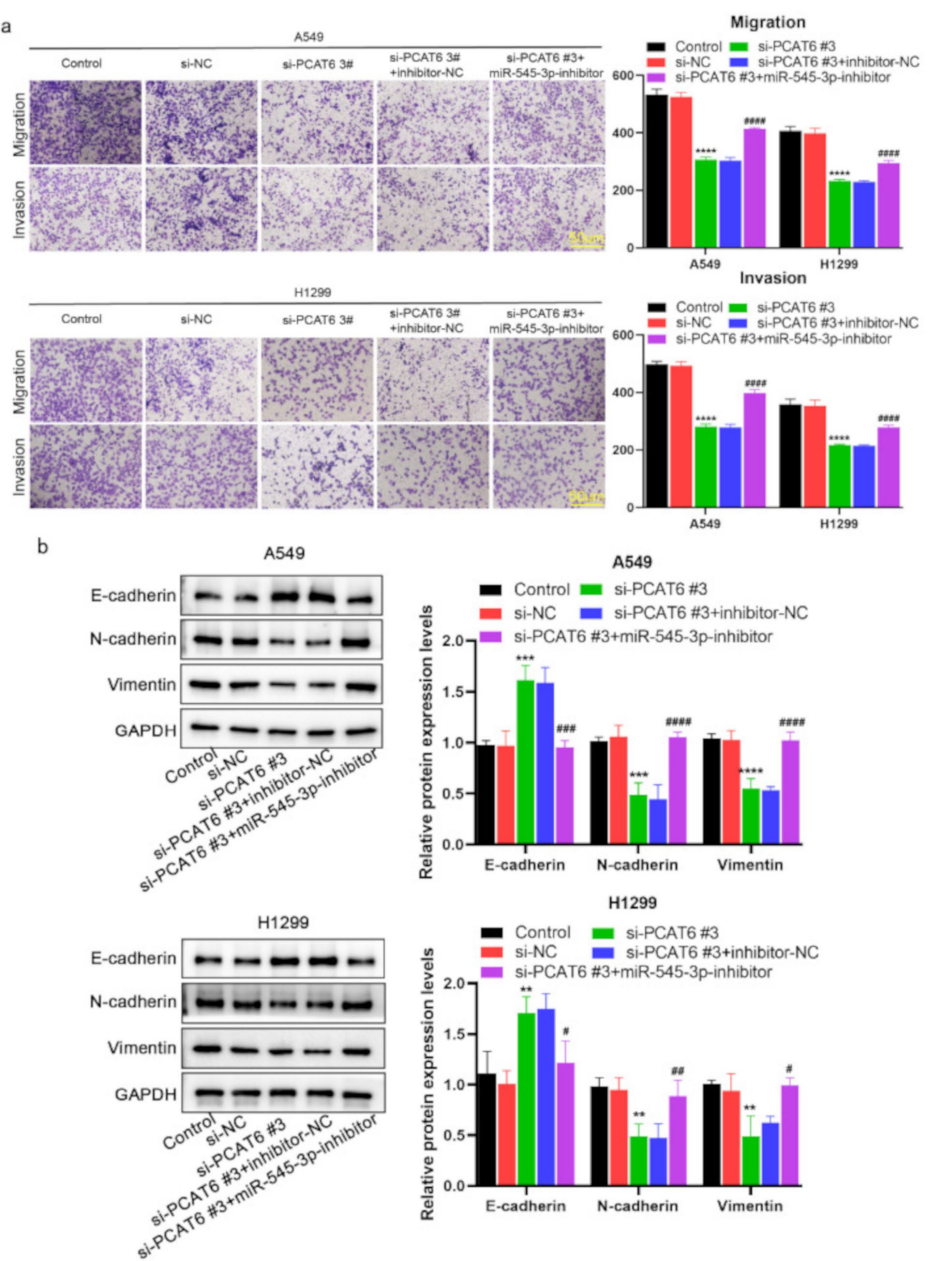
MiR-545-3p inhibitor partially abolished lncRNA PCAT6 silencing inhibition of EMT, migration and invasion (a) Transwell assay was used to detect the migration and invasion of two lung adenocarcinoma cells. (b) Detection of EMT-related protein levels by WB assay. *P < 0.05, **P < 0.01, ***P < 0.001 vs. si-NC group; #P < 0.05, ##P < 0.01, ###P < 0.001 vs. si-PCAT6#3 + inhibitor-NC group

To further demonstrate that lncRNA PCAT6 regulates LUAD malignancy through its effects on miR-545-3p, we transfected miR-545-3p inhibitors into LUAD cells to reduce miR-545-3p expression. By using CCK-8 and EdU assays, we observed that knockdown of lncRNA PCAT6 markedly suppressed cell growth, but cultured LUAD cells displayed increased cell growth when co-transfected with an miR-545-3p inhibitor. This finding clearly indicates that miR-545-3p inhibitor partially reversed the influence of lncRNA PCAT6 silencing on cell proliferation (Fig. 4a-b). Similar findings were observed in a wound healing (Fig. 4c) and transwell assays (Fig. 5a). In Western blotting revealed that silencing of lncRNA PCAT6 up-regulated E-cadherin, whereas the levels of N-cadherin and Vimentin were significantly down-regulated. When si-PCAT6 and miR-545-3p inhibitor were co-transfected into A549 and H1299 cells, the miR-545-3p inhibitor partially abolished the impact of lncRNA PCAT6 silencing on these results (Fig. 5b). In summary, we conclude that lncRNA PCAT6 facilitates LUAD cell progression through the regulation of miR-545-3p.

### Prediction and validation of miR-545-3p potential targeted genes

**Fig. 6:**
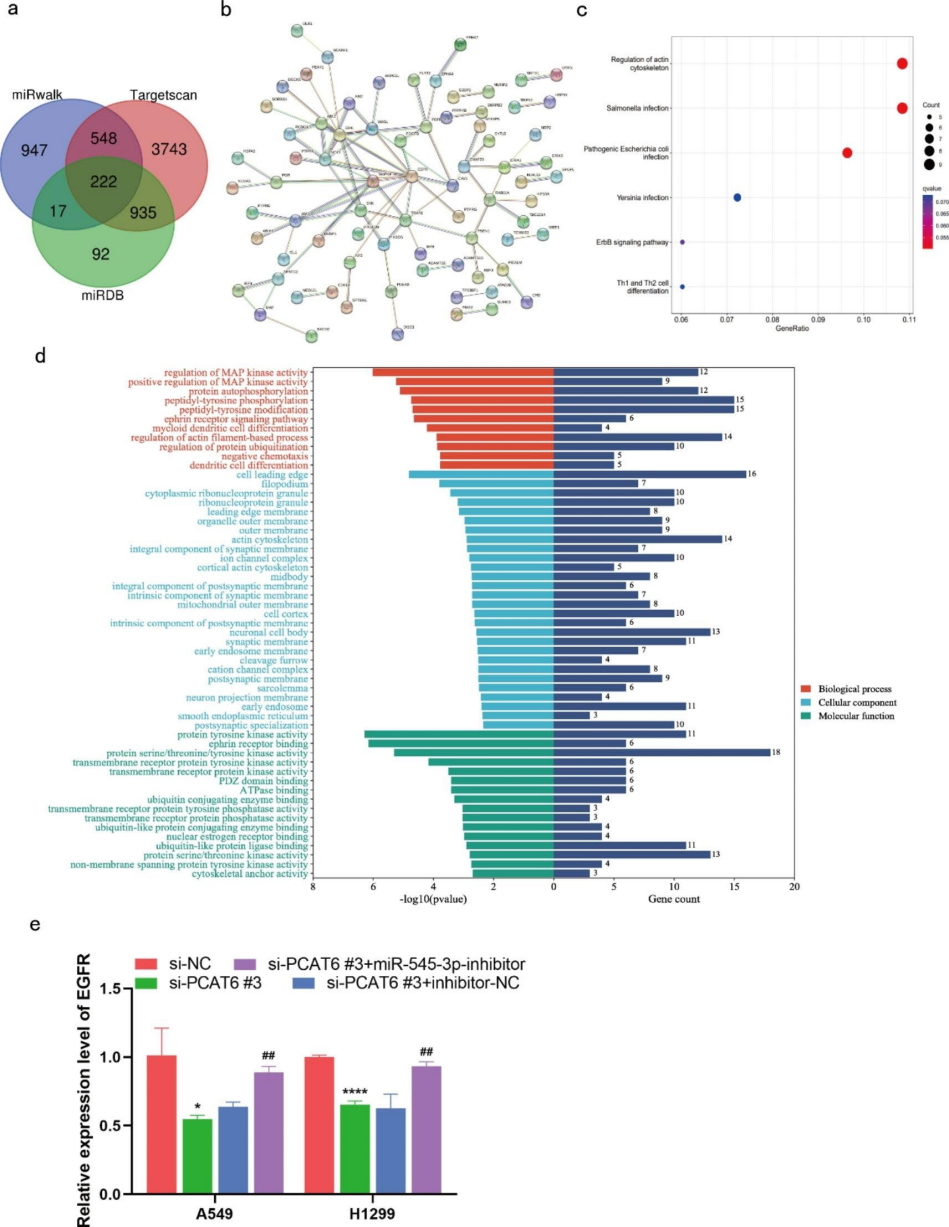
Predicted target mRNA of miR-545-3p (a)Schematic illustration to show the overlapping of the target mRNAs of miR-545-3p, as predicted by miRwalk, TargetScan and miRDB. (b) protein-protein interaction (PPI) network of the predicted target mRNAs of miR-545-3p. (c) KEGG enrichment analysis of the potential target mRNAs of miR-545-3p. (d) GO term enrichment for the potential target mRNAs of miR-545-3p. (e) The expression of EGFR was detected by RT-qPCR. *P < 0.05, **P < 0.01, ***P < 0.001 vs. si-NC group; #P < 0.05, ##P < 0.01, ###P < 0.001 vs. si-PCAT6#3 + inhibitor-NC group

Three databases including RDB (http://www.mirdb.org/), miRwalk (http://mirwalk.umm.uni-heidelberg.de/), and Targetscan (http://www.targetscan.org/vert_71/) were used to predict target genes of miR-545-3p. The results of these database searches resulted in identification of a total of 222 potential targets (Fig. 6a). These 222 targets were used to construct a protein-protein interaction (PPI) network through the STRING database, and the results suggest a close interaction between these proteins. The protein network was imported into Cytoscape for core gene analysis, and the results suggested that EGFR was located at the center of the PPI network, as well as being a core gene transcript targeted by miR-545-3p (Fig. 6b). Further, the results of GO enrichment analysis uncovered three biological aspects, including biological process (BP), cell composition (CC), and molecular function (MF). In terms of biological process, the functions associated with the core gene mainly include regulation of MAP kinase activity and protein autophosphorylation. In terms of cell composition, the components associated with core genes are principally those that control cell leading edge and filopodium. In terms of molecular function, the main core gene-related molecular functions were protein serine/threonine/tyrosine kinase activity, and protein serine/threonine kinase activity (Fig. 6d). The results of KEGG pathway analysis showed that the signaling pathways associated with core genes mainly included ErbB signaling pathway and Th1 and Th2 cell differentiation (Fig. 6c). Subsequently, RT-qPCR indicated that that EGFR was down-regulated after lncRNA PCAT6 knockdown, and that this effect partially recovered after transfection with si-PCAT6 and miR-545-3p inhibitor (Fig. 6e).

## Discussion

The study of LUAD is increasingly focused on the mechanisms of LUAD oncogenesis and the development of new therapeutic approaches to target LUAD. Research has found that lncRNAs are participate in LUAD initiation and development, and may act as possible prognostic factors and therapeutic targets [21, 22]. LncRNA PCAT6 is associated with pro-tumorigenic effects and has been implicated as having a role in lung cancer. For instance, a recent study found that silencing of the lncRNA PCAT6 inhibits macrophage M2 polarization and that NSCLC cell-derived exosomes facilitate macrophage M2 polarization by trafficking lncRNA PCAT6. Macrophage M2 polarization could also facilitate the EMT process and NSCLC metastasis via by the lncRNA PCAT6/miR-326/KLF1 axis[23]. In addition, it was found that lncRNA PCAT6 inhibited gefitinib resistance in NSCLC by up-regulating IFNARS through a sponging of miR-326 [24]. However, the mechanism of how lncRNA PCAT6 promotes LUAD malignancy is not fully understood. Our study found that lncRNA PCAT6 was highly expressed in both LUAD tumors and cultured cells. Further, we document that lncRNA PCAT6 facilitating EMT, and increases growth, migration, and invasion of cultured LUAD cells. Furthermore, we identified miR-545-3p as a putative target of lncRNA PCAT6 by using bioinformatic prediction tools.

MiR-545-3p has attracted increasing attention in human diseases and cancer. A recent study has found that down-regulation of miR-545-3p can facilitate the onset and progression of oral squamous cell carcinoma [25] and when miR-545-3p expression is upregulated, the progression of pancreatic ductal adenocarcinoma is suppressed [26]. In lung cancer, lncRNA FAM83H-AS1 was inversely associated with miR-545-3p expression in lung cancer samples and lncRNA FAM83H-AS1 regulates the HS6ST2 protein by targeting miR-545-3p. Inhibition of miR-545-3p promotes cell invasion and the level of HS6ST2 protein [27]. Moreover, circular RNA hsa_circ_0007580 can promote NSCLC malignancy. Specifically, dual luciferase reporter gene assays showed miR-545-3p was the target of hsa_circ_0007580 [20]. Our research similarly confirmed the decreased expression of miR-545-3p in LUAD.

We also used several tools to predict the downstream target genes of miR-545-3p. The results indicated a total of 222 potential target genes were uncovered. Enrichment analysis revealed that the differential genes were significantly enriched in the regulation of MAP kinase activity, protein autophosphorylation and tyrosine kinase activity. Further, EGFR was found to have the highest connectivity among the potential target genes of miR-545-3p. Epidermal growth factor receptor (EGFR) is a transmembrane protein with cytoplasmic kinase activity that transmits important growth factor signals from the extracellular environment to the intracellular compartment and is the second most important oncogenic factor in non-small cell lung cancer (NSCLC) [28]. EGFR expression is commonly increased in LUAD, and EGFR mutation correlates with lung cancer malignancy, and has become an important target for the treatment of many cancers, especially in lung cancer [29]. Inger et al. reported that NSCLC patients with EGFR mutations display longer times until disease recurrence or death, and increased overall survival when compared to patients with no EGFR mutations [30]. Likewise, Tada et al. also suggested that gemcitabine-targeted EGFR therapy prevents early recurrence in lung cancer patients [31]. Both studies suggest that EGFR has an important role in the mechanism of lung cancer development. In the present study, we also found that EGFR was up-regulated after reducing miR-545-3p abundance in LUAD cell lines, which revealed competitive endogenous RNA (ceRNA) mechanisms controlling EGFR may be a potential complementary strategy for lung cancer treatment.

To date, the ceRNA hypothesis has been extensively studied, and lncRNAs regulate tumor occurrence and development by interacting with miRNAs as ceRNAs [32]. For example, lncRNA FAM83A-AS1 up-regulates ZEBI and ZEB2 through ceRNA acting as miR-141-3p, this affects the EMT process in LUAD [33]. LncRNA HOTAIR can serve as a molecular sponge for miR-20a-5p and markedly facilitates breast cancer malignant progression through activation of HMGA2 protein expression [34]. Our findings indicate that lncRNA PCAT6 was highly expressed in LUAD samples and cells, while miR-545-3p expression was downregulated. Therefore, we propose a ceRNA model for lncRNA PCAT6 and miR-545-3p in LUAD. Using a combination of bioinformatic predictions and dual luciferase reporter gene assays, we proved that lncRNA PCAT6 affects miR-545-3p activity as a ceRNA. MiR-545-3p binding sites on lncRNA PCAT6 sequences were further identified and a miR-545-3p inhibitor blunted the impact of lncRNA PCAT6 silencing on EMT, migration, invasion and growth. Moreover, these findings support the idea that lncRNA PCAT6/miR-545-3p might be a new therapeutic target for LUAD treatment.

Several limitations should be acknowledged in the present study. First, our study explored the role of lncRNA PCAT6/miR-545-3p pathway in LUAD by silencing lncRNA PCAT6 and miR-545-3p expression; however, we did not examine potential effects stemming from overexpression of lncRNA PCAT6 and miR-545-3p on LUAD cell function. Second, although we confirmed that EGFR is regulated by miR-545-3p through bioinformatic predictions and RT-qPCR assays, the direct binding of miR-545-3p and EGFR requires further investigation. Third, no animal experiments were performed to examine the effect of lncRNA PCAT6 expression in LUAD using an in vivo model system.

## Conclusion

Overall, lncRNA PCAT6 expression is increased in LUAD tissues as well as cultured cells. Down-regulation of lncRNA PCAT6 inhibits EMT, migration, invasion and proliferation of cultured LUAD cells. LncRNA PCAT6 can act as a molecular sponge for miR-545-3p, and that this promotes LUAD development. The discovery of lncRNA PCAT6/miR-545-3p network may offer new insights for the diagnosis and treatment of LUAD.

## Electronic supplementary material

Below is the link to the electronic supplementary material.

Supplementary Material 1
