# Correction: LncRNA PCAT6 promotes proliferation, migration, invasion, and epithelial-mesenchymal transition of lung adenocarcinoma cell by targeting miR-545-3p

**DOI:** 10.1007/s11033-025-10859-8

**Published:** 2025-08-21

**Authors:** Chuyi Yang, Hongyu Huang, Yongpeng Li, Ting Zhuo, Lu Zhu, Chenyang Luo, Yanbin Wu, Shouming Qin

**Affiliations:** 1 Department of Pulmonary and Critical Care Medicine, The First Affiliated Hospital of Guangxi Medical University, Nanning, China; 2 Department of the urology, Guangxi Medical University Cancer Hospital, Nanning, China

**Correction to: Molecular Biology Reports (2023) 50:3557–3568**

10.1007/s11033-023-08259-x

In this article, Fig. 5a appeared incorrectly and have now been corrected in the original publication. For completeness and transparency, the old incorrect versions are displayed below.

The original article has been corrected.

Incorrect version of Fig. 5:

**Fig. 5:**
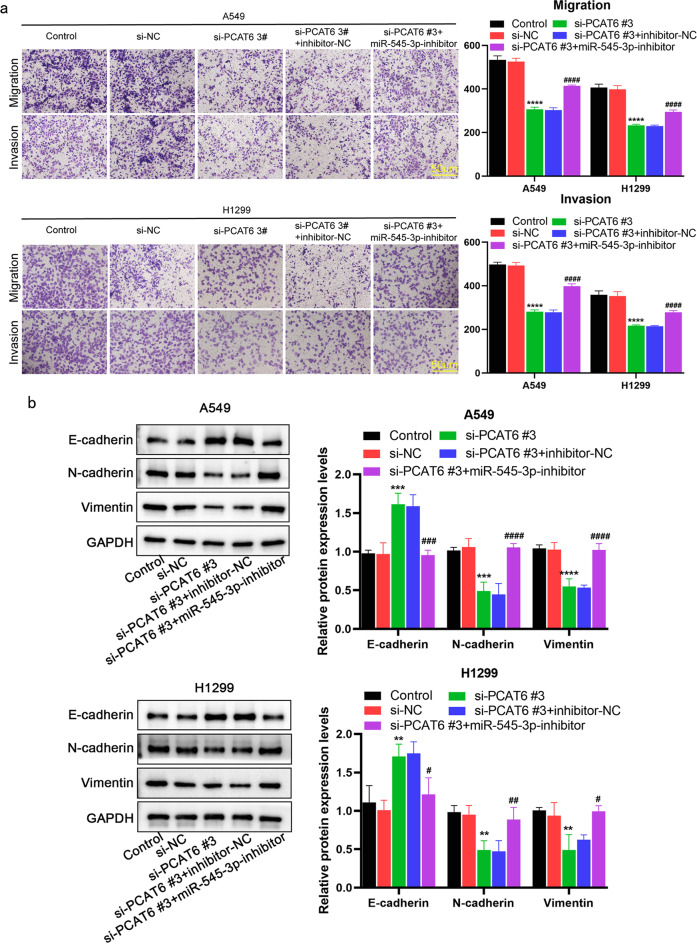
MiR-545-3p inhibitor partially abolished lncRNA PCAT6 silencing inhibition of EMT, migration and invasion (**a**) Transwell assay was used to detect the migration and invasion of two lung adenocarcinoma cells. (**b**) Detection of EMT-related protein levels by WB assay. *P < 0.05, **P < 0.01, ***P < 0.001 vs. si-NC group; #*P* < 0.05, ##*P* < 0.01, ###*P* < 0.001 vs. si-PCAT6#3 + inhibitor-NC group

Correct version of Fig. 5:

**Fig. 5:**
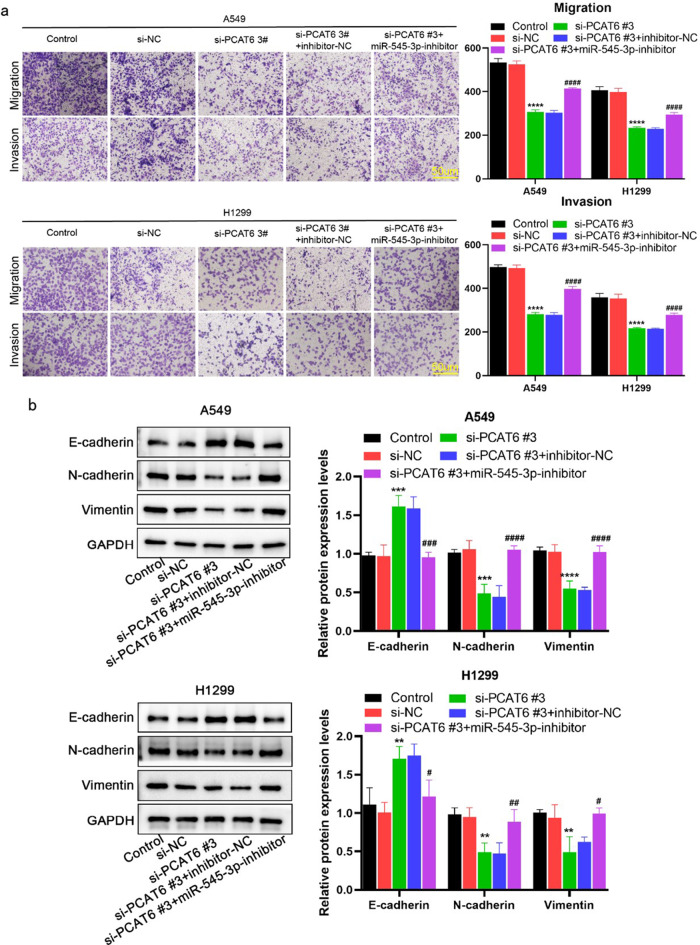
MiR-545-3p inhibitor partially abolished lncRNA PCAT6 silencing inhibition of EMT, migration and invasion (**a**) Transwell assay was used to detect the migration and invasion of two lung adenocarcinoma cells. (**b**) Detection of EMT-related protein levels by WB assay. *P < 0.05, **P < 0.01, ***P < 0.001 vs. si-NC group; #*P* < 0.05, ##*P* < 0.01, ###*P* < 0.001 vs. si-PCAT6#3 + inhibitor-NC group

