# Supplementary material for: LncRNA PCAT6 promotes proliferation, migration, invasion, and epithelial-mesenchymal transition of lung adenocarcinoma cell by targeting miR-545-3p
Source: Mol Biol Rep. 2023 Feb 14;50(4):3557–68. doi: 10.1007/s11033-023-08259-x (PMC10042954; doi:10.1007/s11033-023-08259-x)
Supplement: Supplementary file 1 — Supplementary Material 1 [file 11033_2023_8259_MOESM1_ESM.pdf]

Ejear Editing

CERTIFICATE OF Premium ENGLISH EDITING

This document certifies that the paper detailed below has been standard edited by Ejear. The logical presentation of ideas and the structure of the paper were also checked during the editing process. The edit was performed by professional editors at Ejear. The intent of the author's message was not altered in any way during the editing process. The quality of the edit has been guaranteed, with the assumption that our suggested changes have been accepted and have not been further altered without the knowledge of our editors.

Wednesday, November 30, 2022

Manuscript Title

LncRNA PCAT6 promotes proliferation, migration, invasion and epithelial mesenchymal transition of lung adenocarcinoma cell by targeting miR 545-3p

Manuscript Authors

Chuyi Yang

Certificate Number

PE2022111760
